# Association of Ankle-Brachial Indices With Limb Revascularization or Amputation in Patients With Peripheral Artery Disease

**DOI:** 10.1001/jamanetworkopen.2018.5547

**Published:** 2018-12-07

**Authors:** Homam Moussa Pacha, Vishnu P. Mallipeddi, Naveed Afzal, Sungrim Moon, Vinod C. Kaggal, Manju Kalra, Gustavo S. Oderich, Paul W. Wennberg, Thom W. Rooke, Christopher G. Scott, Iftikhar J. Kullo, Robert D. McBane, Rick A. Nishimura, Rajeev Chaudhry, Hongfang Liu, Adelaide M. Arruda-Olson

**Affiliations:** 1 Department of Cardiovascular Medicine, Mayo Clinic and Mayo Foundation, Rochester, Minnesota; 2 Department of Health Sciences Research, Mayo Clinic and Mayo Foundation, Rochester, Minnesota; 3 Division of Vascular Surgery, Department of Surgery, Mayo Clinic and Mayo Foundation, Rochester, Minnesota; 4 Division of Primary Care Medicine and Center of Translational Informatics and Knowledge Management, Mayo Clinic and Mayo Foundation, Rochester, Minnesota

## Abstract

**Importance:**

The prevalence and morbidity of peripheral artery disease (PAD) are high, with limb outcomes including revascularization and amputation. In community-dwelling patients with PAD, the role of noninvasive evaluation for risk assessment and rates of limb outcomes have not been established to date.

**Objective:**

To evaluate whether ankle-brachial indices are associated with limb outcomes in community-dwelling patients with PAD.

**Design, Setting, and Participants:**

A population-based, observational, test-based cohort study of patients was performed from January 1, 1998, to December 31, 2014. Data analysis was conducted from July 15 to December 15, 2017. Participants included a community-based cohort of 1413 patients with PAD from Olmsted County, Minnesota, identified by validated algorithms deployed to electronic health records. Automated algorithms identified limb outcomes used to build Cox proportional hazards regression models. Ankle-brachial indices and presence of poorly compressible arteries were electronically identified from digital data sets. Guideline-recommended management strategies within 6 months of diagnosis were also electronically retrieved, including therapy with statins, antiplatelet agents, angiotensin-converting enzyme inhibitors or angiotensin-receptor blockers, and smoking abstention.

**Main Outcomes and Measures:**

Ankle-brachial index (index ≤0.9 indicates PAD; <.05, severe PAD; and ≥1.40, poorly compressible arteries) and limb revascularization or amputation.

**Results:**

Of 1413 patients, 633 (44.8%) were women; mean (SD) age was 70.8 (13.3) years. A total of 283 patients (20.0%) had severe PAD (ankle-brachial indices <0.5) and 350 (24.8%) had poorly compressible arteries (ankle-brachial indices ≥1.4); 780 (55.2%) individuals with less than severe disease formed the reference group. Only 32 of 283 patients (11.3%) with severe disease and 68 of 350 patients (19.4%) with poorly compressible arteries were receiving 4 guideline-recommended management strategies. In the severe disease subgroup, the 1-year event rate for revascularization was 32.4% (90 events); in individuals with poorly compressible arteries, the 1-year amputation rate was 13.9% (47 events). In models adjusted for age, sex, and critical limb ischemia, poorly compressible arteries were associated with amputation (hazard ratio [HR], 3.12; 95% CI, 2.16-4.50; *P* < .001) but not revascularization (HR, 0.91; 95% CI, 0.69-1.20; *P* = .49). In contrast, severe disease was associated with revascularization (HR, 2.69; 95% CI, 2.15-3.37; *P* < .001) but not amputation (HR, 1.30; 95% CI, 0.82-2.07; *P* = .27).

**Conclusions and Relevance:**

Community-dwelling patients with severe PAD or poorly compressible arteries have high rates of revascularization or limb loss, respectively. Guideline-recommended management strategies for secondary risk prevention are underused in the community.

## Key Points

**Question:**

Are ankle-brachial indices associated with limb outcomes in community-dwelling patients with peripheral artery disease as identified by computational approaches applied to electronic health records?

**Findings:**

In this cohort study of 1413 community-dwelling patients, peripheral artery disease was identified and limb outcomes were retrieved by computational approaches applied to electronic health records. Ankle-brachial indices of 1.4 or greater were associated with limb amputation and ankle-brachial indices less than 0.5 were associated with limb revascularization.

**Meaning:**

Severe disease and poorly compressible arteries detected by ankle-brachial indices were associated with adverse outcomes in community-dwelling patients with peripheral artery disease; ascertainment of peripheral artery disease phenotypes and outcomes by computational approaches applied to electronic health records is feasible.

## Teaser

This population-based cohort study evaluates whether ankle-brachial indices identified through electronic health records among community-dwelling patients with peripheral artery disease are associated with limb revascularization or amputation.

## Introduction

The prevalence of patients with peripheral artery disease (PAD) is high and increasing worldwide with morbidities that include the need for limb revascularization or amputation.^1,2,3,4,5,6^ Previous studies regarding the rate of limb outcomes have been limited to registry studies^7,8^ and select patient subgroups, including those with critical limb ischemia (CLI),^9,10,11,12,13,14^ prior amputation, or revascularization.^15,16,17^ Moreover, the potential role of noninvasive evaluation by lower extremity ankle-brachial indices (ABIs) for risk assessment and the rates for amputation or revascularization in nonselected patients with PAD from the community have not been established to date.

The 2016 practice guidelines of the American College of Cardiology and the American Heart Association recommend use of secondary prevention therapy to reduce adverse cardiovascular and limb outcomes for patients with PAD.^18^ However, prior studies have demonstrated low rates of adherence to these recommendations, including use of antiplatelet agents, statins, angiotensin-converting enzyme (ACE) inhibitors or angiotensin-receptor blockers (ARBs), and smoking abstention.^18,19,20,21^ The primary objective of the present study was to evaluate whether noninvasive ABI metrics identify patients from the community at high risk for limb revascularization or amputation using computational approaches applied to electronic health records (EHRs) that retrieve PAD phenotypes and limb outcomes. We also sought to evaluate the use of guideline-recommended strategies for secondary prevention for community-dwelling patients with PAD.

## Methods

### Study Design

**Figure 1.**
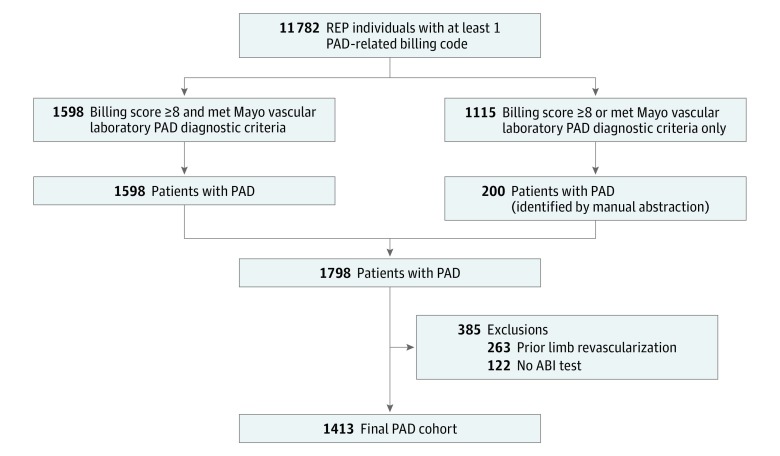
Study Design Patients with peripheral artery disease (PAD) were identified by previously validated electronic algorithms and the medical records linkage system of the Rochester Epidemiology Project (REP). Excluded were those who underwent limb revascularization procedures prior to the date of study entry or did not undergo noninvasive arterial evaluation during the study period. The final study cohort comprised 1413 community-dwelling residents with clinically diagnosed PAD confirmed by noninvasive arterial evaluation. ABI indicates ankle-brachial index.

A test-based community cohort of 1798 patients with PAD diagnosed from January 1, 1998, to December 31, 2011, was assembled and followed up for a median length of 6.3 years. Patients with PAD were identified by previously validated electronic algorithms and the medical records linkage system of the Rochester Epidemiology Project.^22^ A detailed description of this method has been previously reported.^23^ We used resources of the Rochester Epidemiology Project^24,25^ to identify persons with at least 1 PAD-related billing code (Figure 1) and applied our previously validated billing code algorithm^22^ to this data set. Individuals with a total algorithm score of 8 or higher who also met the diagnostic criteria of the vascular laboratory at Mayo Clinic, Rochester, Minnesota, were considered patients with PAD.^22^ A subset of patients had either a billing score of 8 or higher only or met Mayo Clinic vascular laboratory diagnostic criteria for PAD. The medical records of all persons in this subset were manually reviewed to ascertain PAD status. The manual records abstraction identified an additional 200 PAD cases, yielding a cohort of 1798 patients with PAD (Figure 1).

The study was approved by the institutional review boards of the participating institutions. All patients agreed to review of their medical records for research, and the institutional review boards waived the need for informed consent. Data were not deidentified. This study followed the Strengthening the Reporting of Observational Studies in Epidemiology (STROBE) reporting guideline.

For the present study, we excluded patients who underwent limb revascularization procedures prior to the date of study entry or did not undergo noninvasive arterial evaluation during the study period. We included patients with PAD who underwent noninvasive arterial evaluation of the lower extremities at the Mayo Vascular Center. The final study cohort comprised 1413 community residents with clinically diagnosed PAD confirmed by noninvasive arterial evaluation.

Health care institutions participating in the Rochester Epidemiology Project provide medical care to the geographically defined population of Olmsted County, Minnesota. These institutions include Mayo Clinic and affiliated hospitals and Olmsted Medical Center and affiliates.^24^ The health information system of the Rochester Epidemiology Project matches the medical records from participating institutions to specific individuals.^24,25^ Electronic data include demographic characteristics, diagnostic codes, procedural codes, and death information. In addition, for each resident, an EHR and paper records were available for review and abstraction.

### Noninvasive Lower Extremity Arterial Evaluation

All patients underwent clinically indicated testing at the noninvasive vascular laboratory of the Mayo Clinic. Systolic blood pressure was measured for each arm over the brachial artery and for the dorsalis pedis and posterior tibial arteries bilaterally using a handheld, 8.3-MHz Doppler probe.^26^ The ABI was calculated as the highest pedal pressure divided by the highest arm pressure. Peripheral artery disease was defined as an ABI of 0.9 or lower at rest or 1 minute after exercise.^18,27^ The presence of poorly compressible arteries (PCAs) was defined as an ABI of 1.40 or higher.^18,26^ Severe PAD was defined as a resting ABI lower than 0.5.^28^ Data from all lower extremity test results were stored in a digital data set and reported in the Mayo EHR. Ankle-brachial index metrics as well as key words for PCAs were extracted from the EHR using automated text searching.^29^ For analysis, we used the lowest ABI of each patient. For patients who had repeated ABI testing, only the first ABI was used.

### Limb Outcomes

Events were defined as limb revascularization or amputation as ascertained by billing codes (*International Classification of Diseases, Ninth Revision–Clinical Modification* [*ICD-9-CM*] and *Current Procedural Terminology, 4th Edition* procedural codes, as presented in eTable 1 and eTable 2 in the Supplement).^22^ Limb amputation was categorized as major or minor; major amputations included above the knee, below the knee, or foot; and toe amputations were classified as minor.^30^ A trained abstractor blinded to the procedural status manually reviewed a random sample of 20 medical records each for patients who underwent limb procedures and for those who did not undergo limb procedures. The κ agreement between billing codes and manual abstraction was calculated to validate these algorithms, and the values were 0.84 (95% CI, 0.67-1.00) with 90% sensitivity and 94% specificity for revascularization and 0.90 (95% CI, 0.77-1.00) with 90% sensitivity and 100% specificity for amputation.

### Myocardial Infarction, Stroke, and All-Cause Mortality

Myocardial infarction at follow-up was defined by the presence of *ICD-9*-*CM* codes for myocardial infarction (410, 410.x, and 410.x0)^31^ after the index date (ie, date of PAD diagnosis). Stroke at follow-up was defined by the presence of *ICD-9-CM* codes for stroke (434 and 436)^32^ after the index date. Only the first myocardial infarction or stroke was used for analysis. The Rochester Epidemiology Project captured death information through multiple sources, including electronic Minnesota state death certificates, and supplemented these data with information from the National Death Index.^24,25^

### Clinical Characteristics

Previously validated electronic algorithms were also used to ascertain hypertension and hyperlipidemia.^33^ Separate electronic algorithms were applied to ascertain the following comorbid conditions: diabetes, chronic kidney disease, history of myocardial infarction, heart failure, and cerebrovascular disease.^34^ Patients with *ICD-9-CM* codes for atherosclerosis with rest pain (440.22), ulceration (440.23), or gangrene (440.24) were classified as having CLI.^16^ Smoking was ascertained by a combination of previously validated electronic algorithms^35^ and manual abstraction of medical records. These conditions were diagnosed prior to or at the index date of PAD diagnosis.

Medications (aspirin, clopidogrel, statins, ACE inhibitors, and ARBs) in use within 6 months of study entry were also identified. We applied electronic algorithms for ascertainment of medications used during 2006 or at which time an electronic system for medication summary became available.^33^ Records of medications used before 2006 were obtained by manual record reviews conducted by a trained abstractor. Guideline-recommended management strategies included therapy with statins, antiplatelet agents, ACE inhibitors or ARBs, and smoking abstention.^18^

### Statistical Analysis

For analysis, patients were categorized by ABI results into the following groups: severe PAD (ABI <0.5), PCA (ABI ≥1.4), and other ABI values (≤0.9 at rest or 1 minute after exercise and ≥0.5). Baseline characteristics are presented as frequencies and percentages for categorical variables and as means (SDs) for continuous variables. Differences in baseline characteristics were tested between groups using the χ^2^ test for categorical variables or analysis of variance for continuous variables. These overall comparisons were followed by pairwise comparisons using the χ^2^ or 2-sample *t* test as appropriate. Patients were censored at death or last known clinical follow-up for time-to-event outcomes of amputation and revascularization, and only the first event was considered for analyses.

The Kaplan-Meier method was used to estimate incidence rates of limb outcomes, and these were compared between groups using the log-rank test. Cox proportional hazards regression methods were used to analyze the association between severe PAD or PCA with time to event outcomes (limb revascularization, amputation, myocardial infarction, stroke, and death) in unadjusted and adjusted Cox regression models; the proportional hazards assumption was tested for the Cox models. Results of these analyses are summarized as hazard ratio (HR) estimates with associated 95% CIs. The association between guideline strategies and limb outcomes was estimated assuming an ordinal trend across the number of strategies followed. Two-sided tests were used and a *P* value of .05 or less was considered statistically significant. Statistical analysis was performed using JMP, version 10, and SAS, version 9.4 (SAS Institute Inc). Data analysis was conducted from July 15 to December 15, 2017.

## Results

### Clinical Characteristics

A total of 1413 residents (633 [44.8%] were women; mean [SD] age was 70.8 [13.3] years) of Olmsted County who underwent noninvasive arterial evaluation and were diagnosed with PAD were identified. Indications for lower extremity arterial evaluation were rest pain in 71 patients (5.0%), ulceration in 288 (20.4%), gangrene in 41 (2.9%), claudication in 503 (36.0%), and other reasons in 576 (40.8%). Some patients had more than 1 indication for evaluation. Table 1 summarizes baseline clinical characteristics of the severe PAD and PCA groups; 350 patients (24.8%) had PCAs and 283 (20.0%) had severe PAD; 780 (55.2%) other patients with PAD composed the reference group. Age and sex distributions were similar; mean (SD) age of the severe PAD group was 72.6 (11.5) years and the PCA group was 73.6 (13.9) years. There were 349 patients (25.0%) with CLI. The prevalence of CLI was higher in those with PCA than in those with severe PAD (40% vs 30%; *P* < .05). Patients with severe PAD (120 [42.4%]) were more likely to experience claudication than were patients with PCA (48 [13.7%]) (*P* < .001).

**Table 1.** Baseline Characteristics

Characteristic	No. (%)				*P* Value	
	Entire Cohort (N = 1413)	Severe PAD (n = 283)	PCA (n = 350)	Other ABI Values (n = 780)^a^	3 Groups^b^	Severe PAD vs PCA^c^
Year of PAD diagnosis		^d^	^d^			
1998-2001	618 (43.7)	158 (25.6)	128 (20.7)	332 (53.7)	<.001	<.001
2002-2006	507 (35.9)	77 (15.2)	127 (25.0)	303 (59.8)		
2007-2011	288 (20.4)	48 (16.7)	95 (33.0)	145 (50.3)		
Age, mean (SD), y	70.8 (13.3)	72.6 (11.5)^d^	73.6 (13.9)^d^	68.9 (13.4)	<.001	.34
Women	633 (44.5)	142 (50.2)	149 (42.6)	342 (43.8)	.12	.06
Hypertension	1140 (80.7)	231 (81.6)^d^	314 (89.7)^d^	595 (76.3)	<.001	.005
Hyperlipidemia	964 (68.2)	189 (66.8)	233 (66.6)	542 (69.5)	.55	.90
Diabetes	596 (42.2)	93 (32.9)	232 (66.3)^d^	271 (34.7)	<.001	<.001
Chronic kidney disease	370 (26.2)	72 (25.4)^d^	149 (42.3)^d^	149 (19.1)	<.001	<.001
Prior myocardial infarction	383 (27.1)	78 (27.6)	111 (31.7) ^d^	194 (24.9)	.06	.26
Heart failure	425 (30.1)	85 (30.0)^d^	154 (44.0) ^d^	186 (23.8)	<.001	<.001
Cerebrovascular disease	528 (37.4)	124 (43.8)^d^	132 (37.7)	272 (34.9)	.03	.12
Current smoking	358 (25.3)	100 (35.3)^d^	34 (9.7)^d^	224 (28.7)	<.001	<.001
Antiplatelet agents^e^	842 (59.6)	190 (67.1)^d^	212 (60.6)	440 (56.4)	.004	.07
Statins^f^	626 (44.3)	124 (43.8)	147 (42.0)	335 (42.9)	.55	.62
ACE inhibitors or ARBs^e^	638 (45.2)	130 (45.9)	198 (56.7)^c^	310 (39.7)	<.001	.009
No. of guideline strategies			^d^			
0	73 (5.2)	11 (3.9)	8 (2.3)	54 (6.9)	<.001	.03
1	295 (20.9)	59 (20.8)	58 (16.6)	178 (22.8)		
2	424 (30.0)	93 (32.9)	98 (28.0)	233 (29.9)		
3	416 (29.4)	84 (29.7)	114 (32.6)	218 (27.9)		
4	189 (13.4)	32 (11.3)	68 (19.4)	89 (11.4)		
Claudication	507 (35.9)	120 (42.4)	48 (13.7)^d^	339 (43.5)	<.001	<.001
CLI	349 (25.0)	84 (29.7)^d^	139 (39.7)^d^	126 (16.2)	<.001	.009
Median follow-up time, (interquartile range), y	6.3 (3.2-10.1)	5.9 (2.8-9.2)^d^	4.4 (1.9-8.0)^d^	7.5 (4.4-10.9)	<.001	<.001
1-y Limb revascularization rates, % (No. of events)^g^						
Patients with CLI	24 (81)^h^	42 (34)^d^^,^^h^	24 (31)^h^	13 (16)	<.001	<.001
Patients without CLI	15 (162)	28 (56)^d^	8 (17)^d^	14 (89)	<.001	<.001

Abbreviations: ABI, ankle-brachial index; ACE, angiotensin-converting enzyme; ARB, angiotensin receptor blockers; CLI, critical limb ischemia; PAD, peripheral artery disease; PCA, poorly compressible arteries.

^a^ Reference group.

^b^ Analysis of variance test for continuous variables, χ^2^ for categorical variables, and log-rank test for revascularization rates.

^c^ Two-sample *t* test for continuous variables, χ^2^ test for categorical variables, and log-rank test for revascularization rates.

^d^ *P* < .05 for severe PAD and PCA vs other ABI.

^e^ Fourteen patients missing data on antiplatelet agents and ACE inhibitors.

^f^ Sixteen patients missing data for statins.

^g^ Percentages are based on Kaplan-Meier method.

^h^ Log-rank *P* < .01 vs without CLI.

Compared with the severe PAD group, individuals with PCA were more likely to have a history of hypertension, diabetes, chronic kidney disease, heart failure, and therapy with ACE inhibitors or ARBs. Individuals with severe PAD were more likely to be active smokers. The proportion of individuals with hyperlipidemia, prior myocardial infarction, and therapy with aspirin or statins was similar for the 2 groups. The proportion of patients using guideline-recommended secondary prevention strategies for PAD was low overall and in both study groups (Table 1). Only 32 of 283 patients (11.3%) with severe disease and 68 of 350 patients (19.4%) with poorly compressible arteries were receiving 4 guideline-recommended management strategies.

### Limb Outcomes for the Overall Study Group

During a median follow-up of 6.3 years (interquartile range, 3.2-9.9 years), 407 individuals underwent revascularization and 160 had amputation. Among the patients who had amputation, 89 amputations (55.6%) were major and 73 (45.6%) minor, with some patients undergoing more than 1 amputation. The 1-year event rate for revascularization was 17.6% (243 events) and the 5-year rate was 27.6% (353 events). The 1-year event rate for amputation was 6.5% (90 events) and the 5-year rate was 10.0% (129 events).

### Limb Revascularization by ABI Subgroup

**Figure 2.**
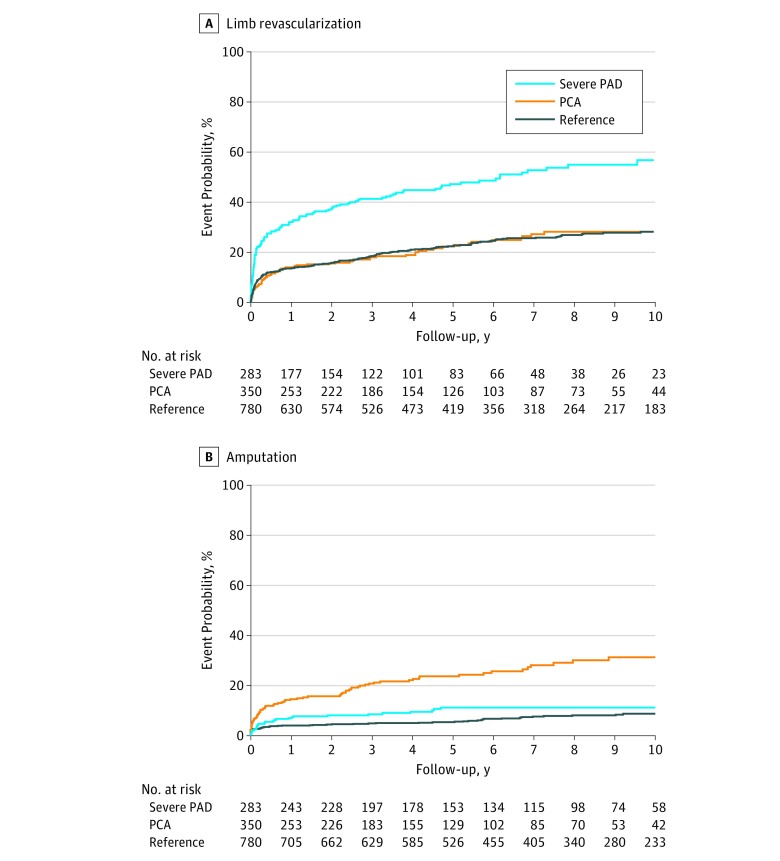
Limb Revascularization and Amputation Event Rates by Ankle-Brachial Index (ABI) Groups A, Patients with severe peripheral arterial disease (PAD) were more likely to undergo limb revascularization than other study groups (log-rank *P* value <.001 across the 3 groups). B, Patients with poorly compressible arteries (PCA) were more likely to undergo limb amputation than the other study groups (log-rank *P* value <.001 across the 3 groups). The reference group included patients with other ABI values.

Patients with severe PAD were more likely to undergo limb revascularization than were those in other study groups (Figure 2A). Severe disease was associated with revascularization (HR, 2.69; 95% CI, 2.15-3.37; *P* < .001). In the severe PAD subgroup, the 1-year event rate for limb revascularization was 32.4% (90 events) compared with a 14.3% (48 events) and 13.7% (105 events) event rate at 1 year in the PCA and reference groups, respectively. Among patients with severe PAD the number of guideline-recommended strategies in use within 6 months of PAD diagnosis did not affect the rates of limb revascularization (HR, 1.02; 95% CI, 0.86-1.22 per strategy; *P* = .78, adjusted for age and sex). Table 2 summarizes the results of Cox proportional hazards regression models for limb revascularization; in these models the presence of severe PAD was associated with increased risk for limb revascularization. By contrast, the presence of PCA was not associated with limb revascularization in any models (HR, 0.91; 95% CI, 0.69-1.20; *P* = .49).

**Table 2.** Cox Proportional Hazards Regression Models for Limb Revascularization by ABI Group

Adjustment	HR (95% CI)		*P* Value	PCA, HR (95% CI)	*P* Value
	Other ABI Values	Severe PAD			
Univariate	1 [Reference]	2.60 (2.08-3.23)	<.001	0.97 (0.75-1.26)	.82
Age and sex	1 [Reference]	2.83 (2.26-3.53)	<.001	1.00 (0.76-1.31)	.98
Age, sex, and year of PAD diagnosis	1 [Reference]	2.92 (2.33-3.66)	<.001	1.01 (0.77-1.32)	.96
Age, sex, and risk factors^a^	1 [Reference]	2.66 (2.12-3.33)	<.001	1.04 (0.78-1.38)	.78
Age, sex, and comorbidities^b^	1 [Reference]	2.79 (2.22-3.49)	<.001	0.98 (0.74-1.28)	.87
Age, sex, and medications^c^	1 [Reference]	2.69 (2.15-3.37)	<.001	0.98 (0.75-1.29)	.92
Age, sex, and CLI	1 [Reference]	2.69 (2.15-3.37)	<.001	0.91 (0.69-1.20)	.49
Age, sex, CKD, diabetes, and CLI	1 [Reference]	2.69 (2.14-3.36)	<.001	0.90 (0.68-1.20)	.49

Abbreviations: ABI, ankle-brachial index; CKD, chronic kidney disease; CLI, critical limb ischemia; HR, hazard ratio; PAD, peripheral artery disease; PCA, poorly compressible arteries.

^a^ Risk factors were current smoking, hypertension, hyperlipidemia, and diabetes.

^b^ Comorbidities were CKD, prior myocardial infarction, heart failure, and cerebrovascular disease.

^c^ Medications were antiplatelet agents, statins, angiotensin-converting enzyme inhibitors, and angiotensin-receptor blockers.

### Limb Revascularization and Critical Limb Ischemia

In the entire study cohort, patients with CLI were noted at follow-up as more likely to have undergone revascularization (eFigure 1 in the Supplement). For patients with CLI, the 1-year event rate for limb revascularization was 24.3% (81 events) compared with a 15.4% event rate (162 events) at 1 year in patients without CLI. Similar to the overall cohort, patients with severe PAD and CLI were more likely to undergo limb revascularization than patients without CLI. Among patients with severe PAD and CLI, the 1-year event rate for limb revascularization was 41.9% (34 events) compared with a 28.4% event rate (56 events) at 1 year in the subgroup of patients with severe PAD without CLI. In the subgroup of patients with PCA, patients with CLI were also more likely to undergo limb revascularization than were patients without CLI. For patients with PCA and CLI, the 1-year event rate for limb revascularization was 23.7% (31 events) compared with an 8.3% event rate (17 events) at 1 year in the subgroup of patients with PCA without CLI. Among patients in the reference group with CLI, the 1-year event rate for limb revascularization was 13.0% (16 events) compared with a 13.7% event rate (89 events) at 1 year in the reference subgroup without CLI.

### Limb Amputation by ABI Subgroup

Patients with PCA were more likely to undergo limb amputation procedures than were patients with severe PAD (1-year rates: 13.9% [47 events] vs 6.6% [18 events]; 5-year rates: 23.1% [69 events] vs 10.5% [26 events]; *P* < .001). Poorly compressible arteries were associated with amputation (HR, 3.12; 95% CI, 2.16-4.50; *P* < .001). Patients with severe PAD were more likely to undergo limb amputation compared with the reference group (Figure 2B) (1-year rate: 3.2% [25 events]; 5-year rate: 4.6% [34 events]; *P* = .05). Poorly compressible arteries were associated with limb amputation in all Cox proportional hazards regression models (Table 3). In contrast, severe PAD was not associated with limb amputation in models adjusted for age, sex, and CLI (HR, 1.30; 95% CI, 0.82-2.07; *P* = .27). Among patients with PCA, the number of guideline-recommended strategies in use within 6 months of diagnosis did not significantly affect the rates of limb amputation (HR, 1.12; 95% CI, 0.91-1.38 per strategy; *P* = .29, adjusted for age and sex).

**Table 3.** Cox Proportional Hazards Regression Models for Limb Amputation by ABI Group

Adjustment	Other ABI Values, Reference	Severe PAD		PCA	
		HR (95% CI)	*P* Value	HR (95% CI)	*P* Value
Univariate	1 [Reference]	1.61 (1.00-2.53)	.04	4.47 (3.16-6.39)	<.001
Age and sex	1 [Reference]	1.74 (1.08-2.74)	.02	4.70 (3.31-6.72)	<.001
Age, sex, and year of PAD diagnosis	1 [Reference]	1.64 (1.03-2.60)	.04	4.80 (3.37-6.84)	<.001
Age, sex, and risk factors^a^	1 [Reference]	1.68 (1.04-2.64)	.03	2.62 (1.81-3.81)	<.001
Age, sex, and comorbidities^b^	1 [Reference]	1.61 (1.00-2.53)	.04	3.89 (2.71-5.64)	<.001
Age, sex, and medications^c^	1 [Reference]	1.65 (1.04-2.62)	.04	4.43 (3.09-6.35)	<.001
Age, sex, and CLI	1 [Reference]	1.30 (0.82-2.07)	.27	3.12 (2.16-4.50)	<.001
Age, sex, CKD, diabetes, and CLI	1 [Reference]	1.33 (0.83-2.12)	.23	1.95 (1.33-2.86)	<.001

Abbreviations: ABI, ankle-brachial index; CKD, chronic kidney disease; CLI, critical limb ischemia; HR, hazard ratio; PAD, peripheral artery disease; PCA, poorly compressible arteries.

^a^ Risk factors were current smoking, hypertension, hyperlipidemia, and diabetes.

^b^ Comorbidities were CKD, prior myocardial infarction, heart failure, and cerebrovascular disease;

^c^ Medications were antiplatelet agents, statins, angiotensin-converting enzyme inhibitors, and angiotensin-receptor blockers.

### Myocardial Infarction, Stroke, and All-Cause Mortality by ABI Subgroup

During median follow-up of 6.3 years (interquartile range, 3.2-9.9 years), 376 individuals had myocardial infarction, 314 had stroke, and 782 died. Myocardial infarction rates were higher in both the severe PAD (HR, 1.62; 95% CI, 1.26-2.09; *P* < .001) and PCA (HR, 1.74; 95% CI, 1.36-2.23; *P* < .001) groups compared with the reference group after adjustment for age and sex. Stroke rates were also higher for PCA (HR, 1.33; 95% CI, 1.01-1.74; *P* = .04) but were not significantly increased for severe PAD (HR, 1.28; 95% CI, 0.96-1.6; *P* = .09). After adjustment for age and sex, death rates were higher in both the severe PAD (HR, 1.38; 95% CI, 1.15-1.64; *P* < .001) and PCA (HR, 1.76; 95% CI, 1.49-2.08; *P* < .001) groups compared with the reference group. Patients with severe PAD and PCA had similar rates of myocardial infarction (HR, 1.08; 95% CI, 0.81-1.43; *P* = .61) and stroke (HR, 1.04; 95% CI, 0.76-1.44; *P* = .81) (eFigure 2 in the Supplement), but the PCA group had a higher rate of death (HR, 1.28; 95% CI, 1.05-1.55; *P* = .01) compared with the severe PAD group after adjustment for age and sex (eFigure 2 in the Supplement).

## Discussion

This study observed that rates of limb amputation and revascularization in community-dwelling patients with PAD are high and results of lower extremity evaluation by noninvasive ABI identify patients with PAD at highest risk for these outcomes. Additional observations included that PCA is strongly and independently associated with amputation and severe PAD is independently associated with revascularization. We also observed that guideline-recommended strategies for secondary risk prevention are underused, confirming a recent national survey.^20^ However, the prior report provided no data on PAD severity and did not capture the longitudinal experience of individual patients.^20^ Moreover, the present study addressed these knowledge gaps in a community setting and included results of lower extremity arterial evaluation by ABIs to assess PAD severity and the presence of PCA as well as longitudinal follow-up.

This study used a computational approach applied to EHRs to identify PAD phenotypes and outcomes of community-dwelling patients evaluated in both inpatient and outpatient settings and was not restricted to survey participants as in prior studies. Hence, the present study added knowledge to prior claims from studies that were limited to summaries of procedural trends or outcomes for patients with PAD but did not assess ABIs for estimation of risk in the community.^36,37,38,39^

This study observed high rates of incident limb amputation or revascularization in community-dwelling patients with PAD compared with prior reports. A previous international registry study noted a lower proportion of patients with PAD who underwent these procedures after 1 year of follow-up.^7^ An important aspect of this prior international registry study was the need for voluntary acceptance for participation, which may have contributed to selection of physicians and patients more likely to adhere to guideline-recommended strategies for secondary prevention than might be expected in community-dwelling residents.^7^ In contrast, in this community-based observational cohort, the voluntary acceptance of physicians was not required. Higher proportions of patients with PAD in the prior registry received guideline-recommended strategies for secondary prevention compared with community residents from the present study.

The study presented herein also included patients with PCA—a cohort excluded from the Reduction of Atherothrombosis for Continued Health registry.^7,8^ We observed the presence of PCA to be strongly and independently associated with limb amputation at follow-up at a median duration of 6.3 years. A prior study limited to patients with CLI also demonstrated that PCA had an independent association with major amputation.^13^ The present study builds on these observations as it included patients who underwent noninvasive arterial evaluation by ABI as well as patients with or without CLI. Hence, the generalizability of our study findings is broader, because it included community-dwelling patients with PAD who did not have CLI.

Our observations suggest the need to increase awareness of the importance of early diagnosis and guideline-directed therapy of PAD, which may promote more timely initiation and optimized secondary prevention strategies to mitigate risk for adverse limb outcomes.^30,31^ In a prior observational study, the adoption of a risk management program promoting use of guideline-recommended strategies for PAD demonstrated fewer limb amputations or surgical revascularization procedures but more endovascular procedures at follow-up.^21^ In another observational study, approximately one-half of the cohort had undergone limb revascularization prior to study entry and, in these patients, use of statins was also associated with lower rates of adverse limb outcomes.^8^

A prior study of patients with PAD who underwent diagnostic angiography or therapeutic endovascular intervention demonstrated that use of 4 guideline-recommended strategies was associated with reduction of mortality and limb revascularization rates but did not affect amputation rates.^19^ In contrast, in the present study, which included patients with PAD from the community and was not restricted to patients who underwent invasive procedures, the use of guideline-recommended strategies did not affect the rates of limb revascularization or amputation. Our observations may be related to the advanced stage of PAD in this cohort, the study design (ie, observational cohort), or insufficient power to evaluate the association between the use of guideline-recommended strategies on limb outcomes given the underuse of these strategies in the study population.

Patients with severe PAD were more likely to experience intermittent claudication and underwent revascularization more often than patients with PCA. Severe PAD may be better recognized clinically by decreased ABI metrics, which may prompt referral for revascularization procedures. However, our observations suggest underrecognition of CLI among patients with PCA. Prior studies have shown that patients with PCA were more likely to have diabetes, to have chronic kidney disease, and to undergo limb amputation.^40,41^ Weinberg et al^42^ have shown by angiography that patients with PCA have high rates of PAD with predilection for the infrapopliteal arteries. In prior studies, for the subset of patients with PCA and CLI, there was angiographic evidence of extensive disease with long lesions and small vessel diameters.^40,41^ Accordingly, our hypothesis is that the mechanism for increased risk for amputation in patients with PCA was likely due to presence of a combination of the following factors: underrecognition of CLI, extensive atherosclerotic disease with long lesions, and small vessel diameter more likely to occur in patients with diabetes and chronic kidney disease.

Our study used computational approaches for identification of a community-based PAD cohort and limb outcomes over time. Using this approach, new knowledge acquired may be linked to clinical decision support, enabling a learning health care system as recommended by a recent Scientific Statement of the American Heart Association.^43,44^ The use of these approaches also offers the potential to rapidly evaluate quality metrics as well as promote increased adherence to guideline-recommended strategies via clinical decision support deployed to EHRs at the point of care.^44^

### Limitations

Limitations of our study included the inability to evaluate multiple procedures over time in the same patient owing to the small number of combined outcomes because most individuals had only 1 limb outcome at follow-up. The majority of revascularization procedures occurred soon after ABI testing and, in many cases, the test results may have prompted the intervention. However, the decisions on patient management were made at the point of care by clinicians and patients and not influenced by the investigators. This study included mostly white, insured individuals who underwent ABI testing at 1 academic institution. Hence, the generalizability of the study findings should be considered limited to individuals with characteristics similar to those of the study cohort.

## Conclusions

Amputation and revascularization rates are high in community-dwelling patients with PAD, and noninvasive vascular testing identifies high-risk individuals, including those with either PCA or severe PAD. Guideline-recommended strategies for secondary risk prevention for patients with PAD are underused in the community setting. Future studies that use big data infrastructure and computational approaches will be able to evaluate the association between clinical decision support deployed to EHRs at the point of care to assess whether rates of adoption of guideline-recommended strategies for secondary prevention in patients with PAD can be improved.

## Notes

Supplement. **eTable 1.** International Classification of Diseases, 9th Revision, Clinical Modification and Current Procedural Terminology-4 Codes for Revascularization **eTable 2.** International Classification of Diseases, 9th Revision, Clinical Modification and Current Procedural Terminology-4 Codes for Amputation **eFigure 1.** Event Rates of Limb Revascularization by CLI Status **eFigure 2.** Event Rates of Myocardial Infarction, Stroke, Death and Combined End-Point by ABI Group
