# Supplementary material for: Association of Ankle-Brachial Indices With Limb Revascularization or Amputation in Patients With Peripheral Artery Disease
Source: JAMA Netw Open. 2018 Dec 7;1(8):e185547. doi: 10.1001/jamanetworkopen.2018.5547 (PMC6324363; doi:10.1001/jamanetworkopen.2018.5547)
Supplement: Supplement. — eTable 1. International Classification of Diseases, 9th Revision, Clinical Modification and Current Procedural Terminology-4 Codes for Revascularization eTable 2. International Classification of Diseases, 9th Revision, Clinical Modification and Current Procedural Terminology-4 Codes for Amputation eFigure 1. Event Rates of Limb Revascularization by CLI Status eFigure 2. Event Rates of Myocardial Infarction, Stroke, Death and Combined End-Point by ABI Group [file jamanetwopen-1-e185547-s001.pdf]

Supplementary Online Content

Moussa Pacha H, Mallipeddi VP, Afzal N, et al. Association of ankle-brachial indices with limb revascularization or amputation in patients with peripheral artery disease. *JAMA Netw Open*. 2018;1(8):e185547. doi:10.1001/jamanetworkopen.2018.5547

eTable 1. International Classification of Diseases, 9th Revision, Clinical Modification and Current Procedural Terminology-4 Codes for Revascularization

eTable 2. International Classification of Diseases, 9th Revision, Clinical Modification and Current Procedural Terminology-4 Codes for Amputation

eFigure 1. Event Rates of Limb Revascularization by CLI Status

eFigure 2. Event Rates of Myocardial Infarction, Stroke, Death and Combined End-Point by ABI Group

This supplementary material has been provided by the authors to give readers additional information about their work.

© 2018 Moussa Pacha H et al. *JAMA Network Open.*

eTable 1. ***International Classification of Diseases, 9th Revision, Clinical Modification*** **and** ***Current Procedural Terminology-4*** **codes for Revascularization.**

Code	Code Type	Description
38.08	ICD-9	Incision of vessel; lower limb arteries
38.18	ICD-9	Endarterectomy; lower limb arteries
38.38	ICD-9	Resection of vessel with anastomosis; lower limb arteries
38.48	ICD-9	Resection of vessel with replacement; lower limb arteries
39.25	ICD-9	Aorto-iliac-femoral bypass
35226	CPT-4	Repair blood vessel, direct; lower extremity
35256	CPT-4	Repair blood vessel, with vein graft; lower extremity
35286	CPT-4	Repair blood vessel, with graft other than vein; lower extremity
35302	CPT-4	Thromboendarterectomy, including patch graft, if performed; superficial femoral artery
35303	CPT-4	Thromboendarterectomy, including patch graft, if performed; popliteal artery
35304	CPT-4	Thromboendarterectomy, including patch graft, if performed; tibioperoneal trunk artery
35305	CPT-4	Thromboendarterectomy, including patch graft, if performed; tibial or peroneal artery, initial vessel
35306	CPT-4	Thromboendarterectomy, including patch graft, if performed; each additional tibial or peroneal artery
35351	CPT-4	Thromboendarterectomy, including patch graft, if performed; iliac
35355	CPT-4	Thromboendarterectomy, including patch graft, if performed; iliofemoral
35361	CPT-4	Thromboendarterectomy, including patch graft, if performed; combined aortoiliac
35363	CPT-4	Thromboendarterectomy, including patch graft, if performed; combined aortoiliofemoral
35371	CPT-4	Thromboendarterectomy, including patch graft, if performed; common femoral
35372	CPT-4	Thromboendarterectomy, including patch graft, if performed; deep (profunda) femoral
35381	CPT-4	Thromboendarterectomy, with or without patch graft; femoral and/or popliteal, and/or tibioperoneal
35454	CPT-4	Transluminal balloon angioplasty, open; iliac
35456	CPT-4	Transluminal balloon angioplasty, open; femoral-popliteal
35459	CPT-4	Transluminal balloon angioplasty, open; tibioperoneal trunk and branches
35470	CPT-4	Transluminal balloon angioplasty, percutaneous; tibioperoneal trunk or branches, each vessel
35473	CPT-4	Transluminal balloon angioplasty, percutaneous; iliac
35474	CPT-4	Transluminal balloon angioplasty, percutaneous; femoral-popliteal
35482	CPT-4	Transluminal peripheral atherectomy, open; iliac
35483	CPT-4	Transluminal peripheral atherectomy, open; femoral-popliteal
35485	CPT-4	Transluminal peripheral atherectomy, open; tibioperoneal trunk and branches
35491	CPT-4	Transluminal peripheral atherectomy, percutaneous; aortic
35492	CPT-4	Transluminal peripheral atherectomy, percutaneous; iliac

© 2018 Moussa Pacha H et al. *JAMA Network Open.*

35493	CPT-4	Transluminal peripheral atherectomy, percutaneous; femoral-popliteal
35495	CPT-4	Transluminal peripheral atherectomy, percutaneous; tibioperoneal trunk and branches
35521	CPT-4	Bypass graft, with vein; axillary-femoral
35533	CPT-4	Bypass graft, with vein; axillary-femoral-femoral
35537	CPT-4	Bypass graft, with vein; aortoiliac
35538	CPT-4	Bypass graft, with vein; aortobi-iliac
35539	CPT-4	Bypass graft, with vein; aortofemoral
35540	CPT-4	Bypass graft, with vein; aortobifemoral
35541	CPT-4	Bypass graft, with vein; aortoiliac or bi-iliac
35546	CPT-4	Bypass graft, with vein; aortofemoral or bifemoral
35548	CPT-4	Bypass graft, with vein; aortoiliofemoral, unilateral
35549	CPT-4	Bypass graft, with vein; aortoiliofemoral, bilateral
35551	CPT-4	Bypass graft, with vein; aortofemoral-popliteal
35556	CPT-4	Bypass graft, with vein; femoral-popliteal
35558	CPT-4	Bypass graft, with vein; femoral-femoral
35563	CPT-4	Bypass graft, with vein; ilioiliac
35565	CPT-4	Bypass graft, with vein; iliofemoral
35566	CPT-4	Bypass graft, with vein; femoral-anterior tibial, posterior tibial, peroneal artery or other distal vessels
35571	CPT-4	Bypass graft, with vein; popliteal-tibial, -peroneal artery or other distal vessels
35582	CPT-4	In-situ vein bypass; aortofemoral-popliteal (only femoral-popliteal portion in-situ)
35583	CPT-4	In-situ vein bypass; femoral-popliteal
35585	CPT-4	In-situ vein bypass; femoral-anterior tibial, posterior tibial, or peroneal artery
35587	CPT-4	In-situ vein bypass; popliteal-tibial, peroneal
35621	CPT-4	Bypass graft, with other than vein; axillary-femoral
35623	CPT-4	Bypass graft, with other than vein; axillary-popliteal or -tibial
35637	CPT-4	Bypass graft, with other than vein; aortoiliac
35638	CPT-4	Bypass graft, with other than vein; aortobi-iliac
35641	CPT-4	Bypass graft, with other than vein; aortoiliac or bi-iliac
35646	CPT-4	Bypass graft, with other than vein; aortobifemoral
35647	CPT-4	Bypass graft, with other than vein; aortofemoral
35651	CPT-4	Bypass graft, with other than vein; aortofemoral-popliteal
35654	CPT-4	Bypass graft, with other than vein; axillary-femoral-femoral
35656	CPT-4	Bypass graft, with other than vein; femoral-popliteal
35661	CPT-4	Bypass graft, with other than vein; femoral-femoral
35663	CPT-4	Bypass graft, with other than vein; ilioiliac
35665	CPT-4	Bypass graft, with other than vein; iliofemoral
35666	CPT-4	Bypass graft, with other than vein; femoral-popliteal
35671	CPT-4	Bypass graft, with other than vein; popliteal-tibial or -peroneal artery
35700	CPT-4	Reoperation, femoral-popliteal or femoral (popliteal)-anterior tibial, posterior tibial, peroneal artery, or other distal vessels, more than one month after original operation
35721	CPT-4	Exploration (not followed by surgical repair), with or without lysis of artery; femoral artery

© 2018 Moussa Pacha H et al. *JAMA Network Open.*

35741	CPT-4	Exploration (not followed by surgical repair), with or without lysis of artery; popliteal artery
35876	CPT-4	Thrombectomy of arterial or venous graft (other than hemodialysis graft or fistula); with revision of arterial or venous graft
35879	CPT-4	Revision, lower extremity arterial bypass, without thrombectomy, open; with vein patch
35881	CPT-4	Revision, lower extremity arterial bypass, without thrombectomy, open; with segmental vein interposition
35883	CPT-4	Revision, femoral anastomosis of synthetic arterial bypass graft in groin, open; with nonautogenous patch graft (e.g. Dacron, ePTFE, bovine pericardium)
35884	CPT-4	Revision, femoral anastomosis of synthetic arterial bypass graft in groin, open; with autogenous patch graft
37184	CPT-4	Primary percutaneous transluminal mechanical thrombectomy, noncoronary, arterial or arterial bypass graft, including fluoroscopic guidance and intraprocedural pharmacological thrombolytic inejction(s); initial vessel
37185	CPT-4	Primary percutaneous transluminal mechanical thrombectomy, noncoronary, arterial or arterial bypass graft, including fluoroscopic guidance and intraprocedural pharmacological thrombolytic inejction(s); second and all subsequent vessel(s) within the same vascular family
37186	CPT-4	Secondary percutaneous transluminal thrombectomy (e.g. nonprimary mechanical, snare basket, suction technique), noncoronary, arterial or arterial bypass graft, including fluoroscopic guidance and intraprocedural pharmacological thrombolytic injections, provided in conjunction with another percutaneous intervention other than primary mechanical thrombectomy
37205	CPT-4	Transcatheter placement of an intravascular stent(s) (except coronary, carotid, and cerebral vessel), percutaneous; initial vessel
37206	CPT-4	Transcatheter placement of an intravascular stent(s) (except coronary, carotid, and cerebral vessel), percutaneous; each additional vessel
37207	CPT-4	Transcatheter placement of an intravascular stent(s) (except coronary, carotid, and cerebral vessel), open; initial vessel
37208	CPT-4	Transcatheter placement of an intravascular stent(s) (except coronary, carotid, and cerebral vessel), open; each additional vessel

Legend: CPT-4= Current procedural Teminology-4; ICD-9= International Classification of Diseases, 9th Revision

© 2018 Moussa Pacha H et al. *JAMA Network Open.*

eTable 2. ***International Classification of Diseases, 9th Revision, Clinical Modification*** **and** ***Current Procedural Terminology-4*** **codes for Amputation.**

Code	Code Type	Description
84.1x	ICD-9	Amputation of lower limb
84.91	ICD-9	Amputation, not otherwise specified
27295	CPT-4	Disarticulation of hip
27590	CPT-4	Amputation, thigh, through femur, any level
27591	CPT-4	Amputation, thigh, through femur, any level; immediate fitting technique including first cast
27592	CPT-4	Amputation, thigh, through femur, any level; open, circular (guillotine)
27598	CPT-4	Disarticulation at knee
27880	CPT-4	Amputation, leg, through tibia and fibula
27881	CPT-4	Amputation, leg, through tibia and fibula; with immediate fitting technique including application of first cast
27882	CPT-4	Amputation, leg, through tibia and fibula; open, circular (guillotine)
27888	CPT-4	Amputation, ankle, through malleoli of tibia and fibula (e.g. Syme, Pirogoff type procedures), with plastic closure and resection of nerves
27889	CPT-4	Ankle disarticulation
28800	CPT-4	Amputation, foot; midtarsal (e.g., Chopart type procedure)
28805	CPT-4	Amputation, foot; transmetatarsal

Legend: CPT-4= Current procedural Teminology-4; ICD-9= International Classification of Diseases, 9th Revision

© 2018 Moussa Pacha H et al. *JAMA Network Open.*

eFigure 1. Event Rates of Limb Revascularization by CLI Status

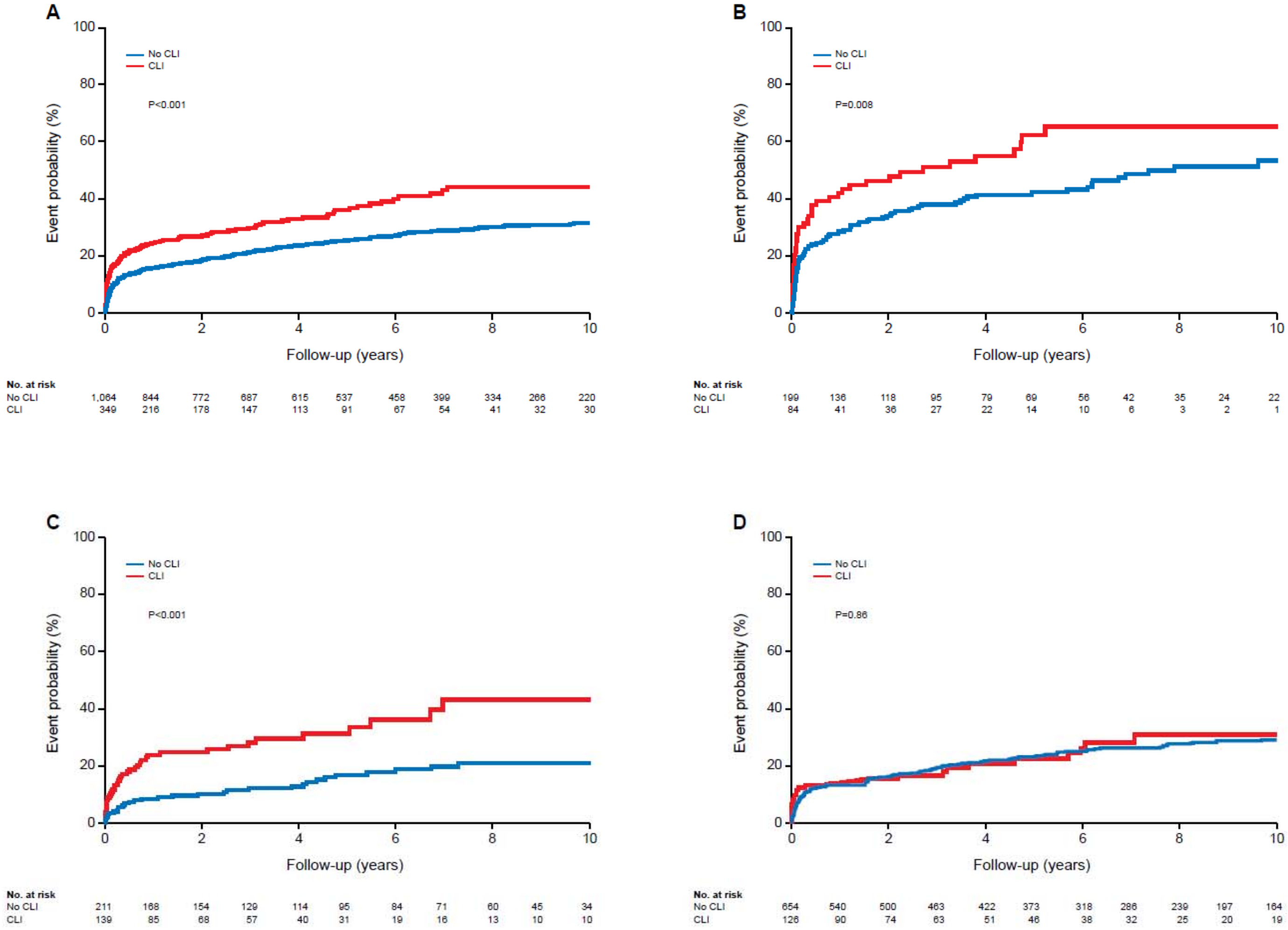

Legend: Patients with CLI were more likely to be revascularized at follow up in the overall cohort (panel A), in the subgroup of patients with severe PAD (panel B); and in the subgroup of patients with PCA (panel C). However, in the reference subgroup (other ABI values) the event-rates were similar for patients with or without CLI (panel D). Log-rank p-values shown. CLI = Critical Limb Ischemia, No = Number

© 2018 Moussa Pacha H et al. *JAMA Network Open.*

eFigure 2 – Title: Event-rates of Myocardial Infarction, Stroke, Death and Combined End-Point by ABI group.

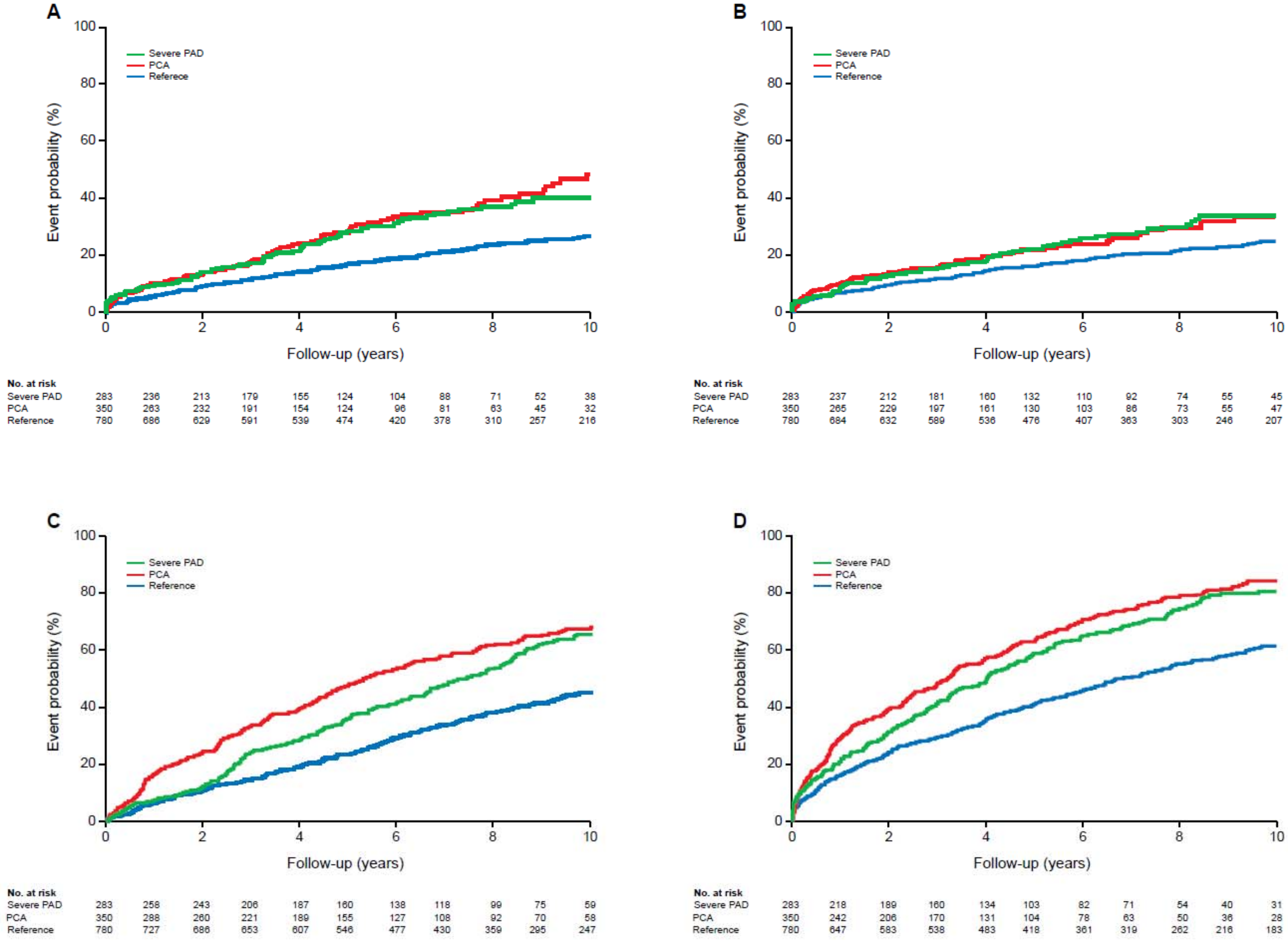

Legend: Patients with severe PAD and PCA had similar rates of myocardial infarction (p = 0.61) and stroke (p = 0.81) but PCA had a higher rate of death (HR = 1.28 (1.05-1.55), p = 0.01) compared to severe PAD, after adjustment for age and sex. No = number; PAD = peripheral arterial disease; PCA = poorly compressible arteries; reference group = other ABI values.

© 2018 Moussa Pacha H et al. *JAMA Network Open.*
